# Design, synthesis and analysis of charged RGD derivatives

**DOI:** 10.6026/97320630019918

**Published:** 2023-09-30

**Authors:** Bonaventure Mujyambere, Subasri Mohanakrishnan, Shoufia Jabeen Mubarak, Hemamalini Vedagiri, Sivasamy Ramasamy, Suja Samiappan

**Affiliations:** 1 Department of Biochemistry, Bharathiar University, Coimbatore, Tamilnadu, India; 2 Department of Bioinformatics, Bharathiar University, Coimbatore, Tamilnadu, India; 3 Department of Human Genetics and Molecular Biology, Bharathiar University, Coimbatore, Tamilnadu, India

**Keywords:** Design, synthesis, analysis, charged RGD derivatives

## Abstract

In the present study, negatively charged N-Biotin-RGD and positively charged C-Biotin-RGD were designed, synthesized, and characterized with docking analysis. The fixation of MDA-MB-231 cells with formalin made their cell surface neutrally charged thus removing the electrostatic interactions between charged biotinylated RGD derivatives and MDA-MB-231 cells. The results of the binding affinity of biotinylated RGD derivatives against MDA-MB-231 cells showed that N-Biotin-RGD had higher binding affinity than C-Biotin-RGD. The cytotoxic effect was analyzed by incubating charged biotinylated RGD derivatives with live MDA-MB-231 cells. MDA-MB-231 cell surface is negatively charged due to high hypersialyliation of polyglycans and Warburg effect. The results of their cytotoxic activities against live MDA-MB-231 cells were found to be electrostatic in nature. C-Biotin-RGD had an attractive interaction with the MDA-MB-231 cell surface resulting in a higher cytotoxic effect. In comparison, N-Biotin-RGD had a repulsive interaction with the MDA-MB-231 cell surface resulting in a lower cytotoxic effect. Hence, positively charged C-Biotin-RGD is a better cytotoxic agent than a negatively charged N-Biotin-RGD against MDA-MB-231 cells.

## Background:

Cancer cells are caused by epigenetic and genetic changes that make the resulting abnormal cells resistant to the normal regulatory checkpoints [1,2] These changes are continuously triggered by the cancer cells in order to take advantage to their ever-changing intracellular activities and their surrounding environment [3]. This includes the need for increased amounts of biomolecules that are involved in metabolism and proliferation [4]. Cancer cells maximize their energy production by adopting aerobic glycolysis as the main source of ATP molecules for highly dividing cancer with lactic acid as its byproduct [5]. Hypersialylation is the addition of sialic acid on glycoconjugate chains which results in promotion of tumor development, inhibition of cellular apoptosis, induction of cell detachment, improvement of cell invasion, enhancement of immune evasion, and induction of metastases [6,7]. The overexpression of lactic and sialic acids is directly proportional to the negative charges on the cancer cell surface. [8]. Computer modeling is one of the leading techniques for designing small biomolecules that are complementary in shape to the binding sites of the intended targets [9,10]. The application of drug design for diagnosis and treatment of various cancers usually focus on biochemical features that are either overexpressed or uniquely expressed in tumor cells [11]. The extracellular receptors are the direct link of communication between the cells and its environment; they are the best options to target as they are easily accessible and can be analyzed straightforwardly [12]. Integrins are extracellular receptors that are involved in most stages of cancer development including tumor development, angiogenesis, cell migration and invasion, anoikis resistance and metastasis [13,14]. Integrins are classified into various subtypes depending on the sequence they recognize and a subset that binding with Arg-Gly-Asp (RGD) motif represents almost half of all the integrins [15]. RGD tripeptide is a zwitterion of arginyl residue on N-terminal end is responsible for the positive charges due to the α-amino group and the guanidine side chain and aspartic acid residue on C-terminal end which provides the negative charge due to carboxyl groups of both the side chain and the C-terminal group [16]. RGD tripeptide has a low cell attachment activity due to its highly flexible conformation when interacting with integrins [17, 18]. However, the blocking of either one of the N- or C-terminal ends resulted in improved cellular activity [19]. This process could be used to improve the binding of RGD towards integrins but also to create positively charged as well as negatively charged derivatives [20]. The presence of biotin is also useful for qualitative as well as quantitative analyses due to the fact that its interaction with streptavidin and its derivatives is among the most stable non-covalent interactions found in nature [21]. The hypotheses of this study mainly are; (i) in-silico drug design could be used to improve the binding affinity of RGD motif on RGD-recognizing integrins; (ii) The conjugation of biotin tags on RGD tripeptide could result in the creation of charged biotinylated RGD derivatives; (iii) Fixation of cells resulting in neutrally charged cancer cells could be used to determine the binding affinities of these biotinylated RGD derivatives; (iv) Live cancer cells could be used to determine the involvement of electrostatic interaction in cytotoxic activities; (v) Structure-activity relationship could determine the best biotinylated RGD derivative for the treatment of breast cancer.

## Materials and Methods:

## Design of charged biotinylated RGD derivatives

The structure of biotinylated RGD tripeptides were drawn using Chemsketch freeware. N-Biotin-RGD was designed by taking N atom of the amino terminal of RGD tripeptide and linking it with C atom of the carboxyl group of biotin. C-Biotin-RGD was designed by linking the last N atom of the hydrazyl group of biotin hydrazide with C atom of carboxyl end of RGD tripeptide.

## Confirmation of charges for biotinylated RGD derivatives:

At the physiological pH, the overall charge of biotinylated RGD derivatives depended on the pKa values of their ionizable groups (Table 1) and was calculated using modified Henderson-Hasselbalch equations:

[1] For the amino terminal: (-NH2) x (10pKa-pH / 10pKa-pH + 1)

[2] For the carboxyl terminal: (-COOH) x (10 -(pKa-pH) / 10 -(pKa-pH) + 1)

[3] For positively charged R group: (R) x (10pKa-pH / 10pKa-pH + 1)

[4] For negatively charged R group: (R) x (10 -(pKa-pH) / 10 -(pKa-pH) + 1)

## Calculation of isoelectric points:

The isoelectric point of an aqueous peptide solution is the pH at which both the positively charged groups and the negatively charged groups of the molecules are at equilibrium. The calculation of pI was done using the following formula:

pI = (pKa1 + pKa2) / 2

Where pKa1 and pKa2 correspond to the values within which the charge of biotinylated RGD derivatives was zero.

## Molecular docking studies:

ITGB1 was downloaded from the Protein Data Bank (PDB ID: 4WJK) and the energy of its 3D structure was minimized using the OPLS3e force field. The selected ligands were prepared using ligprep in Schrodinger Maestro 11.8. After docking at default settings, the lowest binding energy which conforms to the best structure of the docked complexes was selected.

## Materials used:

Arg-Gly-Asp (RGD) tripeptide, Biotin-NHS, Biotin-hydrazide, Cellulose acetate membrane (MWCO = 500 Da), a magnetic biodialyzer, 1ethyl-3-dimethylaminopropyl carbodiimide hydrochloride (EDC) were purchased (Sigma Aldrich, India). DMEM, fetal bovine serum (FBS), bovine serum albumin (BSA), Penicillin-Streptomycin, Phosphate buffer saline (PBS) and Tween-20 were purchased (HiMedia, India).

## MBA-MD-231 cell culture:

MBA-MD-231 cells were obtained from NCCS (Pune, India) and were cultured in High Glucose DMEM containing 10% FBS, and 1% Penicillin-Streptomycin at 37oC, under 5% CO2 and 95% humidity.

## Synthesis of N-Biotin-RGD:

RGD peptide solution (2 mg in 1 ml of PBS) was mixed with biotin-NHS solution (20 mg in 1 ml of DMSO) and incubated overnight at 4°C. The synthesized derivative was purified using a bio dialyzer [22].

## Synthesis of C-Biotin-RGD:

RGD peptide solution (5 mg in 1 ml of 0.1M MES at pH 5.5) was mixed with biotin hydrazide solution (13 mg in 1 ml of DMSO), then 250 µl of the EDC solution was added. The mixture was incubated overnight at room temperature under constant agitation. The synthesized derivative was purified using a biodialyzer [23].

## Binding affinityy assay:

MDA-MB-231 cells were cultured overnight in 96-well plate. The cells were washed, fixed, blocked and the sample solutions were added and the plate was incubated overnight at 4°C. The cells were stained with Streptavidin-HRP and then incubated with TMB solution. The optical densities were read at 590 nm and their relative binding affinities were determined [24].

## Cytotoxicity assay:

MDA-MB-231 cells were incubated overnight in 96-well plate. The media was removed, the sample solutions were added and the plate was incubated for 24 hours. MTT solution was added, followed by DMSO and the optical densities were read at 540 nm [25]. The cell death percentage was calculated using the following formula:

Cell death % = [1 - (OD of treated cells / OD of control cells)] x 100

Where, OD refers to the optical density at 540 nm.

## Statistical analysis:

All experiments were done in triplicate and were expressed as Mean ± SD. Statistical comparison of mean values was performed using ANOVA with p ≤ 0.05 considered statistically significant.

## Structure-activity relationship analysis:

The SAR analysis was performed by comparing the the charge of each biotinylated RGD derivatives with their cytotoxic activities against MDA-MB-231 cells.

## Results and Discussion:

## Design of charged biotinylated RGD derivatives:

**Figure 1:**
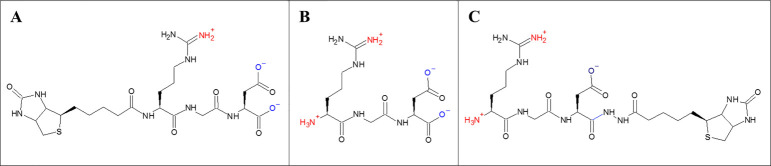
Charged biotinylated RGD derivatives

The designing of biotinylated RGD derivatives was done by adding biotin on N-terminal end of RGD tripeptide to form N-biotinylated RGD derivative (N-Biotin-RGD) while biotin hydrazide was added on C-terminal end of RGD tripeptide to form C-biotinylated RGD derivative (C-Biotin-RGD) (Figure 1).

## Confirmation of the net charges of biotinylated RGD derivatives:

The charges of biotinylated RGD derivatives was found structurally and empirically by finding the sum of all the charges present on each derivative. The overall charge of C-Biotin-RGD was +1, while N-Biotin-RGD has an overall charge of -1 whereas RGD tripeptide was 0 (Figure 1; Table 2).

## Isoelectric potential of biotinylated RGD derivatives:

At the physiological pH, the results showed that N-Biotin-RGD was acidic while C-Biotin-RGD was basic whereas RGD tripeptide was slightly neutral (Table 3).

## Docking of biotinylated RGD derivatives against ITGB1:

**Figure 2:**
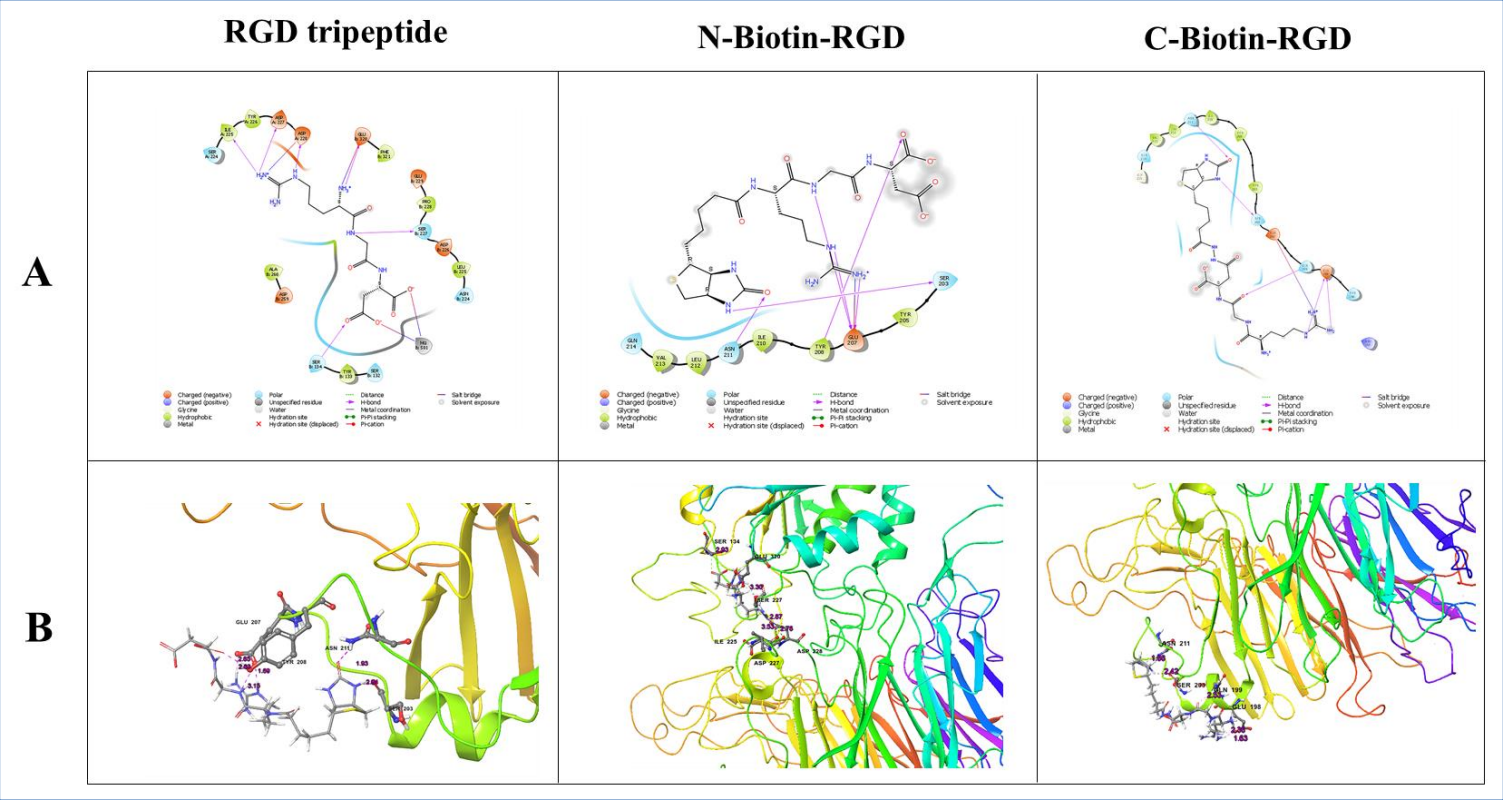
Docking of biotinylated RGD derivatives against ITGB1

The molecular docking results in 2D structures showed the involvement of different amino acids for each ligand against ITGB1 while 3D images showed that each ligand-receptor interaction had its own unique conformation [126]. The bonds formed were unique to each interaction so are the amino acids which were involved in the bond formation (Figure 2; Table 4).

## Synthesis of N-Biotin-RGD:

**Figure 3:**
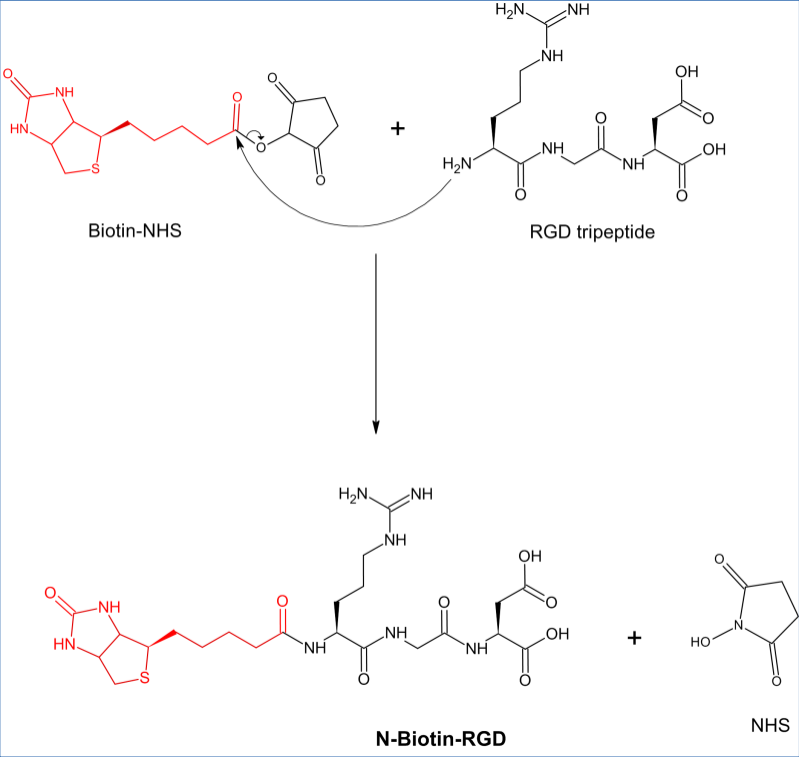
Synthesis of N-Biotin-RGD

The synthesis of N-Biotin-RGD was achieved after the formation of amide bond between amino group of RGD tripeptide and the carboxyl group of biotin (Figure 3).

## Synthesis of C-Biotin-RGD:

**Figure 4:**
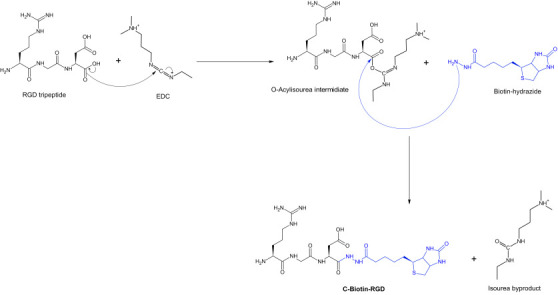
Synthesis of C-Biotin-RGD

The synthesis of C-Biotin-RGD was done in two steps. First step is the activation of the carboxyl group of RGD tripeptide by EDC which resulted in the formation of an unstable O-acylisourea. Second step resulted in the formation of C-Biotin-RGD after the interaction between biotin hydrazide andthe unstable O-acylisourea (Figure 4).

## Binding affinities of biotinylated RGD derivatives against fixed MBA-MD-231 cells:

**Figure 5:**
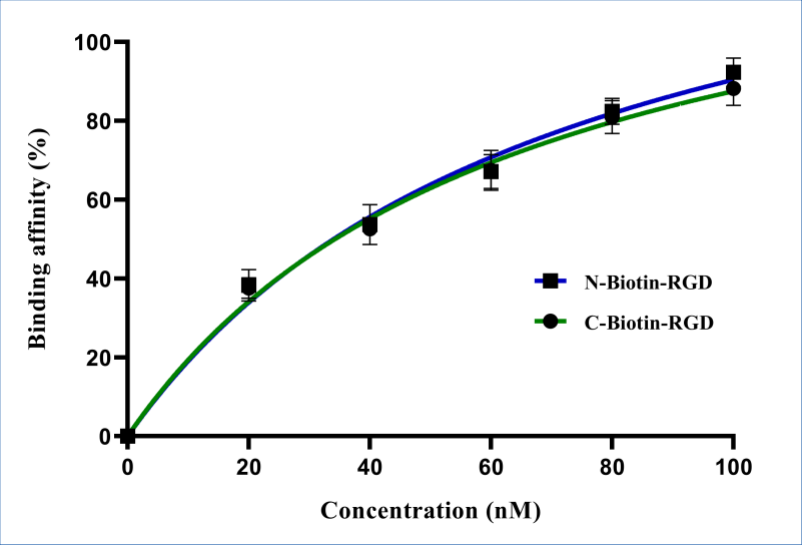
Binding affinities of biotinylated RGD derivatives against fixed MBA-MD-231 cells

The fixation of MDA-MB-231 cells removed negative charges through the creation of methylene bridges by crosslinking cell surface proteins. The relative binding affinities of biotinylated RGD derivatives were used to determine which derivative had higher affinity towards the receptors of MDA-MB-231 cells. The results show that N-Biotin-RGD had the higher binding affinity than C-Biotin-RGD (Figure 5). There was a similarity in strength between the binding affinity and the binding energies predicted with docking analysis [27].

## Cytotoxicity assay:

**Figure 6:**
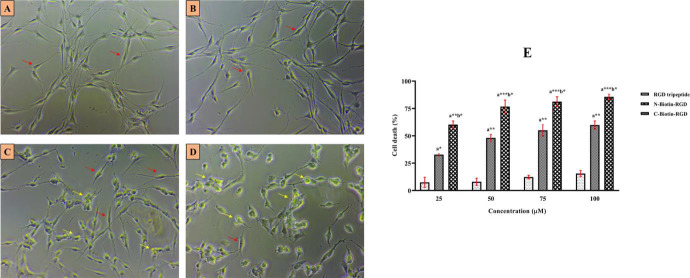
Cytotoxicity assay

The ability to induce cell death of charged biotinylated RGD derivatives was done using live MDA-MB-231 cells. The results confirmed C-Biotin-RGD to be a better cytotoxic agent with an IC50 value of 13.1 ± 2.43 µM than N-Biotin-RGD with IC50 values of 47.58 ± 5.43 µM (Figure 6). Biotinylated RGD derivatives had more improved cytotoxic effects than RGD tripeptide due to the presence of biotin tags which stabilize the conformation of RGD motif [28].

## Structure-activity relationship analysis:

The relationship between the type of charges of biotinylated RGD derivatives and their cytotoxic activities against MDA-MB-231 cells was analyzed by comparing the pI with IC50 values. After IC50 calculations, it was observed that positively charged C-Biotin-RGD had higher cytotoxic effect than negatively charged N-Biotin-RGD. The comparison of pI values concluded C-Biotin-RGD to be basic while N-Biotin-RGD was acidic (Table 5). The comparison of relative binding affinity and cytotoxic activities with isoelectric points may suggest the involvement of the electrostatic interaction when cells were alive and ligand-receptor interactions were the cancer cells were fixed with formalin.

## Conclusion:

Data showed the importance of in-silico studies in designing and testing molecular prospects before their analysis in laboratory settings. The charges created by biotinylation of the end terminals of RGD tripeptide resulted in a positively charged C-Biotin-RGD and a negatively charged N-Biotin-RGD. Even though ligand-receptor interactions may involve electrostatic interactions between them, here the term electrostatic interactions was used for ionic interactions between the ligands and the cancer cell surface. Ionic interactions are stronger and act at a longer distance compare to other intermolecular bonds. Thus, they would be more effective than ligand-receptor interactions in biological activities where both are supposed to be involved. According to our study, the involvement of electrostatic interactions shows that integrin inhibition is not the main inhibitor of cell viability [29]. This is confirmed by comparing the binding affinities of biotinylated RGD derivatives with their cytotoxic activities respectively. N-Biotin-RGD had higher binding affinity and lower cytotoxic activity while C-Biotin-RGD had lower binding affinity and higher cytotoxic activity [30]. Although further studies are required, with the pave of these present findings, our work provided an evidential possibility for correlating the charges of a drug candidate and their effectiveness as cytotoxic agents.

## Figures and Tables

**Table 1:** pKa values of N- and C-terminal residues of RGD tripeptide

**Amino acid**	**pKa**		
	(-COOH)	(-NH_2_)	R group
Arginine (R)	2	9	12.5
Aspartic acid (D)	2	9	3.9

**Table 2:** Calculation of the charges of biotinylated RGD derivatives

**Ionizable groups**	**Guanidine side chain**	**Carboxyl terminal end**	**Amino terminal end**	**Carboxyl side chain**	**Net charge calculation**
pKa and pH	12.5	2	9	3.9	7.4
Formulae for calculation of charges	10^pKa-pH^	10^-(pKa-pH)^	10^pKa-pH^	10^-(pKa-pH)^	Sum total of all charges at pH 7.4
	10^pKa-pH^+ 1	10^-(pKa-pH)^+ 1	10^pKa-pH^ + 1	10^-(pKa-pH^) + 1	
RGD tripeptide	1	-1	1	-1	0
N-Biotin-RGD	1	-1	NA	-1	-1
C-Biotin-RGD	1	-1	1	NA	1
NA - Not Applicable

**Table 3:** pI values of biotinylated RGD derivatives

**RGD tripeptide and its derivatives**	**Net charges between certain pH values**					**pI values**
	**0 - 2.0**	**2.0 -3.9**	**3.9 - 9.0**	**9.0 - 12.5**	**12.5 - 14**	
RGD tripeptide	2	1	0	-1	-2	6.45
N-Biotin-RGD	1	0	-1		-2	2.95
C-Biotin-RGD	2		1	0	-1	10.75

**Table 4:** The docking analysis of biotinylated RGD derivatives against ITGB1

**Ligand**	**Bonds involved**	**Involvement of**	**involvement of**	**Involvement of ionizable Side Chains**	**Docking Score**	**Glide Energy**
		**amino acid Residues**	**Biotin ring**			
RGD tripeptide	10	SER B:227	-	SER B:134	-7.53	-69.819
		GLU B:320 (2)		ILE A:225		
		MG B:501		ASP A:227		
				ASP A:228 (2)		
				MG B:501		
N-Biotin-RGD	7	GLU207	SER203	GLU207 (3)	-5.86	-39.601
		TYR208	ASN211			
C-Biotin-RGD	6	GLN 199	SER203	GLU198 (2)	-6.427	-45.498
			ASN211	GLU202		

**Table 5:** Structure-activity relationship analysis of biotinylated RGD derivatives

**Biological activities**	**RGD tripeptide**	**N-Biotin-RGD**	**C-Biotin-RGD**
Binding affinity	-	+ + +	+ +
Cytotoxic activities	-	+ +	+ + +
The biological activities were symbolized with "-" for negative effects; "+" for the positive effects; "+ +" for more positive and "+ + +" for the most positive effects.
